# “I’m trying to take the lead from my child”: experiences Parenting Young Nonbinary Children

**DOI:** 10.1186/s13034-024-00807-y

**Published:** 2024-09-12

**Authors:** Noah Sweder, Lucinda Garcia, Fernando Salinas-Quiroz

**Affiliations:** 1 Eliot-Pearson Department of Child Study and Human Development (EPCSHD), Tufts University, Medford, USA; 2 School of Arts and Sciences, EPCSHD, Tufts University, 105 College Avenue, 02155 Medford, USA

**Keywords:** Nonbinary, Nonbinary children, Transgender and gender diverse (TGD) youth, Transgender and nonbinary (TNB) youth, Parental support, Gender affirmation, Cisnormativity, Qualitative research, Reflexive thematic analysis

## Abstract

**Background:**

While research has emphasized the importance of parental support for LGBTQIA + youth wellbeing, there remains limited understanding of parental experiences with nonbinary children, particularly those prepubescent. This study aimed to explore how parents of nonbinary children ages 5–8 learn to support their child’s identity, examining initial reactions, emotional processes, supportive behaviors, societal responses, and associated challenges and rewards.

**Methods:**

A qualitative study was conducted using Reflexive Thematic Analysis (RTA) within a framework of ontological relativism and epistemological constructivism. Nine parents of nonbinary children aged 5–8 from the Northeastern United States participated in semi-structured interviews lasting 60–80 min. Questions explored various aspects of parenting nonbinary children, including the child’s gender identity, parental feelings, experiences sharing the child’s identity, and challenges and rewards of raising a gender-diverse child. The research team, comprising individuals who identify as trans, genderqueer, and nonbinary, employed collaborative coding and thematic development.

**Results:**

Four main themes were constructed: (1) *Parents hear and support their child’s nonbinary identity*, this theme highlights immediate acceptance and efforts parents make to affirm their child’s gender; (2) *Parents learn about ways cisnormative society harms their child*, here, parents recognize the societal pressures and barriers their children face; (3) *Parents take significant and proactive steps to affirm their child*, this theme documents the actions parents take to support their child in environments that invalidate their identity; and (4) *Gender is just one aspect of who my child is*, this theme reflects on parental insights of gender as just one part of their child’s overall personhood.

**Conclusions:**

This study provides insights into the experiences of parents supporting young nonbinary children, emphasizing the importance of affirming expressed identity, the parent-child relationship, and proactive support in navigating cisnormative societal structures. Findings highlight the transformative experience of parenting nonbinary children, with parents often challenging their own preconceptions of gender and coming to more nuanced understandings. These results can inform supportive interventions and policies for nonbinary children and their families, and we hope to contribute to a growing body of research that shifts narratives towards joy, resilience, and community in trans and nonbinary experiences.

## Background

Over the past three decades, cultural discourse around ‘intensive parenting’ [1] has emphasized the need to dedicate significant time, energy, and resources to raising children in a way that prioritizes their ability to communicate needs and self-advocate [2]. While current literature highlights the importance of parental support for the wellbeing of LGBTQIA + youth [3–5], there is still very little research focused on the experiences of parents of trans and nonbinary (TNB) children [6].

In this context, Hidalgo et al. [7] propose a gender-affirmative model of supportive parenting that views genderqueernes not as a disorder but as a culturally influenced variation shaped by biology, development and socialization. This model acknowledges that gender is fluid rather than binary and suggests that the struggles children experience with identity often stem from societal prejudices like transphobia and the institution of cisnormativity, an element of the social, political and economic system in our society designed to force individuals into a gender and sex binary, while punishing behavior and expression that deviates from it. Legal and medical professionals are primary enforcers of cisnormativity. Despite being society’s supposed experts on gender and sex, as a field they fail to acknowledge the self-determination of gender and conceptualize sex as a spectrum [8]. This begins with the assessment of genitals at birth and the 'corresponding' gender assignment [9, 10].

Amidst the ongoing discourse on gender fluidity, we are witnessing a significant amount of anti-transgender violence. Trans people–especially TNB children– are increasingly becoming the targets of political campaigns, fearmongering, and public debates that challenge their right to exist. In 2023, the number of anti-trans legislation in the U.S. surged dramatically, from 174 (26 passed) to 604 (87 passed), more than tripling the record set the year before. By July 2024, 635 bills had been introduced into state legislatures, with 123 active and 47 passed [11]. These laws aim to exclude TNB children from accessing healthcare, updating legal documents to reflect their gender, using appropriate bathroom facilities, and participating in school activities such as sports and clubs [12].

Given these vicious attacks on children, the role of the family in supporting their TNB child has become increasingly critical. Research consistently demonstrates that family dynamics significantly impact the psychological health of LGBTQIA + youth, with supportive environments providing a buffer against stigma, and promoting overall wellness [3–5]. Recent longitudinal studies underscore that while TNB youth face increased risks for adverse mental health outcomes, their wellbeing improves significantly within supportive family contexts [13]. Central to this support is the acceptance and understanding of nonbinary identities and expressions, including the adoption of gender-neutral language by family members, which enhances the child’s perception of acceptance from their parents [14–16].

The sharing of pronouns is becoming increasingly popular in the U.S. and beyond, including Western countries like the UK and Canada, as well as parts of Asia, especially among younger generations and progressive circles [17–21]. A 2020 Pew Research Center survey found that over half of Americans are aware of pronouns that are not ‘she/her’ and ‘he/him’, with younger people being more likely to use them. This trend reflects a growing global awareness of gender existing beyond the binary, as well as the importance of validating these identities through inclusive language. As awareness and popularity of gender diversity grows, it has become popular to share pronouns in introductions and email signatures [22], and transphobic people have been attempting to politicize pronouns in an effort to villainize TNB people. The adoption of gender-neutral pronouns, like the singular ‘they’, which gained recognition when Merriam-Webster named it the word of the year in 2019, underscores an ongoing challenge to binary norms.

In this paper, we define ‘nonbinary’ as an umbrella term encompassing individuals who self-identify as a gender outside the gender binary, and/or does not identify as always and completely being just a man or a woman, recognizing gender as existing along a spectrum. Various expressions of nonbinary identities are present, such as identifying as both a boy and a girl, experiencing gender as fluid or fluctuating, feeling a partial connection to one gender without fully aligning with it (known as demigender), embracing multiple gender identities, adhering to two-spirit traditions rooted in Indigenous cultures, adopting gender concepts from ‘unrelated domains’ (known as xenogender), or ‘lack of’ a gender [23]. While terms like ‘trans’ and ‘nonbinary’ originate from Western contexts, they strive to encompass a diverse and evolving spectrum of gender identities. It is important to note that while many nonbinary individuals may identify as trans, not all do.

For nonbinary people, recognition and affirmation is deeply intertwined with language use. Employing gender-affirming language—such as neutral labels, pronouns, and grammatical structures—in environments like schools, workplaces, healthcare facilities, and the home, is crucial for encouraging “self-definition,” as well as “visibility and understanding” of nonbinary identities [24]. In Budge and colleagues [14]’ five-year study of families with a TNB member, pronouns were best understood by the end of the research period. Their findings suggest that conducting regular family check-ins on gender identity, pronouns, and gender expression can significantly enhance family members’ understanding of the individual’s needs. Matsuno and colleagues [25] found that support can be further conceptualized as advocating for the child’s rights across various settings, expressing love verbally and nonverbally, and actively seeking community and professional resources for the growth of both the parent and the child.

Some studies have documented that initial parental reactions to their child’s TNB identity such as shock, fear, and worry, often hinder their acceptance and support [26, 27]. These emotional barriers, compounded by cisnormativity and transphobia in society, may lead to reluctance in acknowledging or understanding TNB identities [28]. Moreover, entrenched beliefs in binary and immutable gender norms further complicate parental efforts to support nonbinary children. In fact, Matsuno and colleagues [4]’ study found that among parents of TNB youth, half of those who were unsupportive had nonbinary children, indicating potentially greater challenges in support within this demographic.

McGuire and colleagues [29] theorized that the presence of a TNB individual in a family leads to other members of the family challenging existing theories about essentialist and social constructionist notions of gender and sexuality. Given that gender is “messy, plural and in constant evolution” [30], these authors argue that describing it requires “dynamic approaches […] that can account for within-person variability over time” ( [29] p.63). This perspective underscores the evolving nature of gender identities within families and highlights the need for flexible mental frameworks that are welcoming to the diverse expressions of nonbinary individuals.

Addressing emotional barriers, transphobia, and cisnormativity necessitates parents being equipped with the knowledge and skills to navigate the social exclusion of nonbinary identities effectively [27, 28]. Facilitators of supportive parental behaviors include building social support networks, and accessing informational resources [4]. Connecting with others, whether online or in person, is crucial for parents of TNB youth, highlighting the importance of making support groups accessible [31]. Exposure to positive portrayals of gender diversity also plays a crucial role in fostering affirming parental attitudes and behaviors [32, 33]. Schools often lack knowledge about gender diversity and inclusive practices suitable for all children, regardless of their gender identity. Therefore, in these environments, parents have to ‘make room’ for their children by informing school staff about their child’s chosen names, pronouns, individual needs, and sometimes even providing basic education on gender identity and diversity [34].

The trans family systems framework, proposed by Robinson and Stone [35], challenges traditional family dynamics by integrating trans identities as an analytical category [36]. This category questions the sex/gender distinction, challenges biological determinism, exposes the production of normativity, and disrupts cisnormative gender practices. The trans family systems approach explores how either investments in or divestments from cisnormativity shape both family interactions and individual experiences. The concept of *cisgender divestments* [35] describes how family members resist cisnormative gender norms to support their gender-diverse children. Actions such as providing a variety of toys, clothes, and activities (i.e., ‘gender buffet’ [37]), and validating TNB identities (i.e., ‘giving gender’ [38]), can be seen as forms of cisgender divestment.

Parents are not only socializing their children, rather, parents are also re-socializing themselves. This means that many caregivers are rethinking their own relationship to gender as they engage in ‘gender-expansive childhood socialization’ [39]. Initially, children are often required to ‘prove’ their identity. Subsequently, these same parents of TNB children often find themselves explaining their children’s gender to others [40]. Many caregivers transition from confusion and uncertainty to ‘pride’ and ‘empowerment,’ becoming advocates for TNB people beyond their children [29]. Learning to affirm one’s TNB child not only enhances family cohesion, but also contributes to a broader divestment from cisnormativity.

The evolution of parental responses to nonbinary identities reflects a broader societal shift from pathologizing to affirming non-normative identities. According to de Bres [41]’ critical review of research on parents of gender-diverse children, early studies from the 1990s and 2000s predominantly took a pathologizing stance toward gender diversity in children, often validating parents’ negative reactions. During the 2010s, research began to shift towards a more affirming perspective, although it still frequently equated the experiences of a ‘parental transition’ to a process of grief [42], continuing a level of pathologization. Hidalgo and Chen [43] assert that parents experience both external stressors (e.g., school discrimination, rejection by family and friends, and verbal abuse) and internal stressors (e.g., negative messages about gender diversity and ‘fitting in’ difficulties) while supporting their nonbinary child’s identity. Parents worry about their child’s well-being in a society that often invalidates gender diversity. Supportive parents might also face stigma and a reduction in social safety, such as losing connections with family, friends, and religious communities. Additionally, they must address their own cisnormative beliefs and navigate barriers in educational and healthcare settings. These challenges can impact their ability to support their nonbinary child [25].

Since the late 2010s, there have been numerous documented stories of parents highlighting their support for TNB children by shielding them from societal stigma, embracing a gender-affirming approach, and normalizing gender diversity in their home and broader community [41]. Recent studies have shown how parents are reframing the narrative of grief, instead focusing on the rewards of parenting a TNB child, which include ‘greater critical awareness’ [44], ‘expanded knowledge’ [45], and ‘personal growth as a parent’ [46]. Abreu et al. [47] describe this shift as “using radical hope to create meaning and purpose for their child’s existence and envisioning positive future possibilities for them” (p.7). Exploring positive experiences in parenting TNB children can counterbalance the traditional focus on challenges, reflecting many parents’ narratives of joy and transformation.

In summary, the literature underscores the critical role of family support in shaping the wellbeing of nonbinary youth, highlighting the transformative power of parental acceptance and understanding. However, existing research predominantly focuses on trans boys and girls, primarily adolescents, and their families. Little attention has been paid to families with young children who identify beyond the gender binary. Our study aims to “contribute something to a rich tapestry of understanding”^1^ by exploring, in a general sense, the experiences of parents of nonbinary children, guided by our research question, ‘How do parents of nonbinary children learn to support their child?’ Specifically, we seek to understand initial reactions to their children sharing their gender, and changes over time, as well as emotional processes, supportive behaviors, societal responses, and the challenges and rewards for parents in this context. By exploring these dynamics, we aim to contribute insights that can inform supportive interventions and policies tailored to meet the needs of young nonbinary children and their families.

### Methodology 

Our study^2^,^3^ was initially conceived by [AUTHOR 3– MASKED] and further developed with [AUTHOR 1– MASKED] during a summer scholars’ program that supports juniors and seniors in conducting ten-week research projects with faculty mentors. [AUTHOR 3– MASKED], a Brown trans, genderqueer, and nonbinary Mexican immigrant who holds a doctoral degree from the largest university in Latin America, also serves as an Assistant Professor at a private ‘little ivy’ university in the U.S. [AUTHOR 1– MASKED] is a white, trans, genderqueer, and nonbinary undergraduate student at the same institution^4^.

The research question for this study was addressed within a paradigmatic framework of ontological relativism and epistemological constructivism. This fully qualitative approach (i.e., ‘Big Q’), combined with Reflexive Thematic Analysis (RTA), fosters a nuanced and reflective research practice. Unlike more structured and positivistic methods, ontological relativism views reality as diverse and shaped by human actions and interactions [48]. Interpretations of reality differ across cultures and contexts, acknowledging that reality is not fixed. This study is based on the idea that people construct meaning from their experiences (constructionism) and express this through their individual perspectives (relativism). Epistemological constructivism posits that knowledge is dynamic and evolves as individuals reflect on their beliefs and experiences, contributing to collective understanding. This qualitative analysis values the symbolic power of language in data collection, recognizing its role in meaning-making [48].

### Recruitment

Parents were eligible to participate in our study if their child identified beyond the gender binary (see page 1), was aged between 3 and 8 years, and lived in the Northeastern U.S. (Connecticut, Maine, Massachusetts, New Hampshire, New Jersey, New York, Pennsylvania, Rhode Island, and Vermont) to make transportation expenses feasible. Prepaid train rides, parking fees, and/or bus fare were provided.

Between July and August 2022, we created a database and contacted organizations supporting LGBTQIA + individuals/families. In October 2022, specialists from the National Institutes of Health Clinical and Translational Science Program (NIH CTSI) launched a one-month Facebook targeted advertising campaign (UL1TR002544) and posted an advertisement on Craigslist from October 2022 to March 2023. Eleven parents completed a Qualtrics questionnaire, and nine met the inclusion criteria (see Table 1), proceeding to the scheduling of an in-person visit. Families received a $50 USD gift card as compensation. Despite efforts to sample a diverse population, seven participating parents were white, one was Asian-white, and one was Asian. Additionally, all were married, lived in the Greater Boston Area, and earned between the categories of $50,000 to $74,999 USD, and $150,000 USD and above, with a mode of $150,000 USD and above. For reference, the median household income in Boston was $81,744 in 2021^5^. Although we desired to interview parents of children ages 3–5, we only recruited those with children ages 5–8.

**Table 1:** Participants

Participant (pronouns)	Parent Race/Ethnicity	Education	Employment	Religion	Child Gender Identity	Child’s age
Adrienne (she/her)	Asian, white	Graduate degree	Unemployed	None	Both a boy and a girl	6.1
Bianca (she/her)	white	College degree	Unemployed	Spiritual but not religious	Not a boy or girl	6
Christine (she/her)	white	Graduate degree	Full Time Work	Other	“Nonbinary trans girl- sometimes a girl, sometimes nonbinary, never a boy”	6.11
Dahlia (she/her)	white	Graduate degree	Part Time Work	Other	“That’s a tricky one”	6.6
Emiko (she/her)	Asian	Graduate degree	Full Time Work	None	Both a boy and a girl	7.5
Fiona (she/her)	white	Graduate degree	Full Time Work	Other	Not a boy or girl	6.11
Grace (she/her)	white	Graduate degree	Full Time Work	None	Not a boy or girl	8.11
Helena (she/her)	white	Graduate degree	Full Time Work	Spiritual but not religious	Changing between two or more genders frequently Sometimes a boy, sometimes a girl, etc.	5.2
Iris (she/her)	white	Some college or a technical degree	Unemployed	Christianity	Not a boy or girl	8.2

* Note: Data regarding parents’ ages were not collected for this study

### Methods: interviews

The semi-structured interview consisted of 10 predetermined questions, with consent documented from all participants. The interviews typically lasted 70 minutes on average, ranging from 60 to 80 minutes. Questions were designed to explore various aspects of parenting children who identify beyond the gender binary, including describing the child’s gender (e.g., ‘How would you describe your child’s gender?’), changes in gender identity over time (e.g., ‘Has their gender changed over time?’), parental feelings towards the child’s gender identity (e.g., ‘How do you feel about them being [preferred term for child’s gender]?’), experiences sharing the child’s gender identity with others (e.g., “How have people in your child’s life reacted to them being [preferred term]?’), as well as the challenges and rewards of raising a gender-diverse child (e.g., ‘What has been the most rewarding part of raising a [preferred term] child?’). The study adhered to ethical standards set by institutional and national research committees, following the principles of the 1964 Helsinki declaration and its subsequent amendments or comparable ethical standards.

### Reflexivity

[AUTHOR 2 - MASKED], a white graduate student who identifies as trans, genderqueer, and nonbinary, transcribed the interviews. In summer 2023, [AUTHOR 3– MASKED], along with [MASKED], conducted a 5-day workshop on Reflexive Thematic Analysis (RTA). Although exclusively mentioning our skin color, gender identities and educational attainment may resemble a ‘brief confessional’ - see the first paragraph of the Method-ology section and [49] -, on the workshop’s first day, each author spent a 4-hour period crafting their own reflexivity statement, dedicating the initial hour and a half to writing and the remaining time to sharing. These statements proved invaluable throughout the analysis.

Under AUTHOR 3’s guidance, who regularly practices this exercise each semester, the other authors were prompted to reflect on their intersecting identities, social privileges, and marginalities. They were asked to consider how these positions influence their perspectives in the research, and how it may affect how they are perceived by others. Additionally, they reflected on how their backgrounds, life experiences, and beliefs shape their worldview. Next, the authors examined their relationship with knowledge, scholarship, and research practice, considering their research training, experiences, and understanding of ‘good quality’ research, as well as institutional pressures including the capitalist demand to ‘publish or perish.’ They also explored their methodological preferences and how these choices impact the research process and outcomes. Finally, AUTHOR 3 encouraged them to revisit reflexivity regarding their identities and experiences in relation to the project and nonbinary children. They considered how their positions intersect with this topic and examined any assumptions about individuals inside and outside the gender binary. These statements, while lengthy, fostered a sense of closeness and safety among the group. Ideally, they would be included here, but due to the constraints of academic publishing and the emphasis on brevity, interested readers can contact the corresponding author for access.

In summary, the three of us identify as trans, genderqueer, and nonbinary individuals, and share the following beliefs: (a) reality and truth are constructed, contingent, and multiple; (b) gender is experienced in a unique way by everyone; (c) nonbinary individuals know who they are [50]; and (d) we prioritize compassion and curiosity over comprehension [51].

### Reflexive Thematic Analysis (RTA)

Thematic Analysis is a method for identifying, analyzing, and interpreting patterns across qualitative data. Reflexive Thematic Analysis (RTA) differentiates itself by valuing “.a subjective, situated, aware and questioning researcher, a reflexive researcher.” ( [48], loc. 1246). We followed the six-phase RTA process: *familiarizing* ourselves with the interviews, engaging in collaborative *coding*,* developing*,* reviewing*,* refining* themes, and finally, producing this *analytic report.*

### Coding

While familiarizing ourselves with the data is an ongoing process with no single approach, coding is structured and systematic. We carefully read each interview at least twice, identifying and labeling segments relevant to our research question (‘How do parents of nonbinary children learn to support their child?’). Not all segments were coded, as codes are “.ultimately guided by your research question and purpose.” ( [48], loc. 2953).

Collaborative coding enhanced our understanding, interpretation, and reflexivity, rather than achieving consensus on codes. The three authors worked together, discussing and reflecting on their ideas and assumptions. Our aim was to “.gain richer or more nuanced insights collaboratively, *not t*o reach agreement on every code.” ( [48], loc. 3028). This structured exploration ensured a thorough analysis for theme development and attempted to safeguard against accusations of *cherry-picking*.

We used an inductive approach with predominantly semantic codes. Pure induction is impossible as “.we bring various perspectives, theoretical and otherwise, to our meaning-making,” and as a result “our engagement with data is never purely inductive” ( [48], loc. 3027). Semantic codes capture expressed meanings, often mirroring participants’ language, unlike latent coding, which prioritizes underlying meanings that are not explicitly stated.

### Thematization (development, revision, and refinement)

Developing initial themes from our codes involved several processes. We explored areas of similar meaning within the data, clustering potentially connected codes into candidate themes, and examining these patterns of meaning. Each cluster was considered independently, in relation to the research question, and within the broader analysis. We recognized that data “do[es] not speak for [itself];” as researchers, we interpret and tell the story of our data [48].

Phase four provided a crucial check on initial theme development through re-engagement with coded data extracts and the entire dataset. This iterative process ensured that our analysis effectively addressed our research question with a compelling narrative, remaining grounded in the data. We ensured each theme captured a distinct core point and offered rich diversity and nuance, verifying that themes were coherent, distinct, and comprehensive.

### Analysis

We constructed four themes that emerged from the accounts of participating parents. The first theme, *Parents hear and support their child’s nonbinary identity*, details how children share their nonbinary identity with their parents and documents parents’ initial reactions and meaning-making processes. The second theme, *Parents learn about ways cisnormative society harms their child*, captures how parents learn about the struggles their child faces living in a cisnormative society as a nonbinary person. The third theme, *Parents take significant and proactive steps to affirm their child*, documents participants taking action to support their child in environments that do not validate their gender identity. The fourth and final theme, *Gender is just one aspect of who my child is*, discusses insights and conclusions drawn by parents about their child, as well as gender identity. These themes are discussed in detail below.

Theme 1: Parents hear and support their child’s nonbinary identity.

Among all parents, a universally reported experience was receiving a clear and straightforward declaration from their children about their nonbinary identity. Some examples of these explicit statements include: “They always say, I’m not a girl or a boy” (Dahlia), “[They] tell me that [they are] both a boy and a girl” (Helena), and “They describe themself as ‘they/them’” (Iris). All parents were told by their child the way they had been referred to and understood thus far was not correct (i.e., as exclusively a girl or a boy).

All but one parent’s reaction to their child informing them of their gender identity was immediate support for their desired changes (e.g., pronouns and name): “‘Can you call me they/them?’ ‘Okay, great’” (Iris); “Awesome! Whatever you want, we will follow you and affirm you” (Fiona); “We just said ‘okay,’ and we did it” (Grace). The overwhelming support from these parents reflects other child-led approaches, placing affirmation of the child’s lived experience at the center of their parenting style, and validating their emotions and desires even without necessarily understanding them [2].

Some parents initially found it challenging to adjust to using their child’s new pronouns or name, but they all made their best effort. Bianca candidly shared that her husband struggled a lot with they/them pronouns, “not for lack of love or trying, [he] just didn’t have [.] much practice”. The time and effort all parents had to spend to retrain their brain to meet the expressed needs of their child follows the previously mentioned movement towards ‘intensive parenting’ [1].

Despite the dominant paradigm of a gender binary in Western society, no parents reported skepticism of the validity of their child’s nonbinary identities. In fact, the majority of the parents were active in divesting from cisnormativity [35], with six parents explicitly making an effort to raise their children in ways that were not constrained by traditional gender norms. Adrienne explained that she was “quite strongly opposed to having a very gendered upbringing,” and Grace shared a similar sentiment, emphasizing that she wanted her children to “play with whatever [toys] they want,” and that she would buy “whatever clothing” for them. The actions described by these parents exemplify forms of cisgender divestment [35] and a movement away from strict ideas of what a child assigned male at birth (AMAB) or assigned female at birth (AFAB) *should* look like or how they *should* act.

Despite the traditional paradigm of a gender binary in society, no parents reported skepticism of the validity of nonbinary identities. In contrast, the majority of the parents valued divesting from cisnormativity [35], with six parents explicitly making an effort to raise their children in ways that were not constrained by traditional gender norms. Adrienne explained that she was “quite strongly opposed to having a very gendered upbringing,” and Grace shared a similar sentiment, emphasizing that she wanted her children to “play with whatever [toys] they want,” and that she would buy “whatever clothing” for them. The actions described by these parents exemplify forms of cisgender divestment [35] and a movement away from strict ideas of what a child assigned male at birth (AMAB) or assigned female at birth (AFAB) *should* look like or how they *should* act.

Additionally, four parents spoke with their child about gender being more than just boy or girl before their child named themselves nonbinary. Adrienne had “a book about gender identity,” and Fiona had “books that have nonbinary characters and just talk about gender identity and expression.” Bianca “read some books where there was they [pronouns] as an option” and “talked about what that meant ‘’ with her child. Grace could not “point to a moment when [they] started talking about gender and pronouns because it’s [always] been incorporated.” Alternatively, a few parents made no mention of proactively sharing gender-diverse stories, with Christine describing their early conversations around gender as “talking about boys and girls […] talking within the binary, because […] that’s sort of the usual thing.”

Overall, the majority of these parents demonstrated active efforts to divest from expectations of cisnormativity, even before learning their child was nonbinary, with some even deconstructing the gender binary through supportive environments that benefit TNB youth [35, 37, 38]. However, not sharing previous efforts to divest from cisnormativity is also a common experience among both the parents in our study, and parents of TNB children in other studies [29, 40]. There is no evidence supporting the absurd notion that home environments can ‘turn children trans’ [52], and the briefly popular theory of ‘rapid onset gender dysphoria’ has been debunked [53]. Conversely, supportive home environments help TNB children freely explore and come to understand themselves ( [23, 52, 53], and actually fosters a ‘stronger attachment’ between parent and child [42].

While all parents supported their child’s gender identity, some still grappled with doubts and concerns along the way. Iris and Grace questioned whether their child understood what they were saying or was simply using they/them pronouns “because [a peer] said it” (Grace). Grace “didn’t know” whether the desired name/pronoun changes “would stick,” a sentiment echoed by Adrienne’s husband. Additionally, there were concerns about whether the child was “transitioning too young.” Christine expressed caution, not wanting to “act too quickly,” or “be seen to be pushing something on the child,” while Emiko mentioned concerns about their child being perceived as “too young” possibly reflecting societal worries. This experience is not uncommon among parents of TNB children [29]. These parents faced a challenging situation; there is no script for raising nonbinary individuals -as we will discuss further-, and currently, there is a widespread fear-mongering targeting parents of TNB children [53]. Despite these doubts and uncertainties, all but one parent in the study immediately affirmed their child. Many parents expressed worries about their child’s future as a nonbinary person in society, but they understood this as a concern to bear with their spouse, not one with which to burden their child.

Two factors which may have helped parents overcome their doubts, or at least put them aside, are their observations of cisgender nonconformity in their children before the children shared their nonbinary identity, and the insistence of the children themselves.

Bianca, Fiona, Emiko, and Iris’s children who were AMAB all enjoyed wearing dresses, an example of physical presentation not stereotypically associated with their assigned gender. Other parents noted verbal expressions: Adrienne’s child would “call themself a boy or a girl […] depending on what [they] felt more like,” while Christine recalled her child, AFAB, stating plainly that they “don’t always feel like a girl.” Therefore, when these children expressed a desire to use different names or pronouns, it did not necessitate a complete overhaul of how the parents understood them. Reflecting on her reactions to her child sharing their nonbinary identity, Grace shared that she “[was not] really surprised,” Adrienne stated that “[she] had often suspected [her child’s] idea of gender was not [rigid],” and Emiko adding that she felt her child “has never cared about [society’s] gender boundaries.”

Multiple parents mentioned that seeing their child “feel strongly” (Bianca) about their gender identity, “even in the face of lots of situations where it would have been easier [to not identify as nonbinary]” (Dahlia), helped affirm their support. Christine noted that her child’s insistence made it “increasingly clear” to her that their gender identity was both “very important to them” and “that it was real,” and Emiko added “[they] have a sense of agency.”

Although the parents may not fully comprehend their child’s identity, they showed respect for their child as a self-advocate and are committed to centering their child’s expressed needs [1, 2]. This can be seen as a way of prioritizing compassion and curiosity over comprehension [51].

Theme 2: Parents learn about ways cisnormative society harms their child.

With that said, aside from a parent who also identifies as nonbinary, the parents lacked prior understanding of the specific needs of a nonbinary child. We are all familiar with parenting scripts tailored for raising cisgender children, reinforced by abundant resources and support networks, both formal and informal. However, the same cannot be said for parents of nonbinary children. Christine shared her experience of researching how to “best support” her child, finding that there “[is not] a lot out there.” The unique nuances and challenges faced by nonbinary children have not been sufficiently discussed or documented to establish rudimentary frameworks or ‘best practices’ [2]. As a result, these parents found themselves having to find other ways to learn about their child’s needs, largely through conversations with them as well as observing their child’s reaction to experiences, for example, Fiona described herself as a “sponge” absorbing so much new information. Despite their affirmation of the child’s gender identity and use of validating language, it became evident to the parents that their support needed to extend beyond the home.

One common experience for parents was observing their child feeling pressure to “[not] rock the boat” by “bucking” (Fiona) societal norms of gender presentation. Fiona’s AMAB child enjoyed wearing dresses and having their nails painted at home, but intentionally wanted both off before going to school. Helena’s AMAB child was generally open about their feminine expression, “I am a girl, or I feel like a girl,” they would say, yet emphasize, “but it’s secret and I only want you and daddy [to know].” Emiko described an interaction where her AMAB child said, “Most people think that I’m a boy, so if I wear a dress, they’re confused.” The parent followed up, asking “Is that what’s stopping you from wearing a dress?” to which the child responded, “A little bit.” Grace reported her child hesitating about sharing their pronouns with their transphobic grandmother, saying “I don’t want my relationship with grandma to suffer because I’m nonbinary.” This really struck Grace, “the kid is eight years old saying that to me.” All the parents with these experiences shared some understanding gained about their child. Helena reflected that her child “has some awareness […] that being nonbinary is different.” Fiona understood that her child “was self-conscious […] about what people would think,” and Emiko, similarly, saw that her child was feeling pressure from a “societal ‘you should do this.’” It became very clear to the parents that their children were keenly aware of cisnormativity and felt pressure to conform.

Parents also observed barriers to inclusion that exist in what are mundane, day-to-day moments for cisgender people. Some reported their children getting frustrated when confronted with the categorizations, including bathrooms, sections in stores, and birthday party goodie bags. Parents also witnessed their children’s worry and frustration in the context of various forms of potential and realized micro- and macro-aggressions, including experiences of minority stress [54]. For instance, not wearing button up shirts in public “because they were worried that they would be called he” (Bianca), deciding it was “not worth it” to attend to an otherwise ideal camp that was separated into ‘boys’ and ‘girls’ (Emiko), feeling like they “have to” wear a pin that says ‘They/Them’ to avoid being misgendered at school (Grace), and frequently correcting people in public (Adrienne, Bianca, Grace), among many other experiences. Bianca best summarizes what all parents come to understand: their children are made “very upset” about the fact that “the world is not set up to include them.”

When these children leave their homes, they confront a world that overwhelmingly does not validate their identity. Through conversations and observed experiences, parents came to understand distress, anger, and shame their children feel at being constantly invalidated, and gained insights into what kinds of support and affirmation their children need.

Theme 3: Parents take significant and proactive steps to affirm their child.

As expanded on in the second theme (*Parents learn about ways cisnormative society harms their child)*, parents came to recognize the importance of their child’s gender being affirmed and that society is not currently set up to do that. These parents observe their child often having to either self-advocate or endure micro- and macro-aggressions. Following the pattern of intensive parenting, these parents felt a strong desire to advocate for and protect their children, “to make things a little smoother for [their] life” (Iris). It is understood that parental support has a strong influence on the mental health and wellbeing of TNB people [14, 15], highlighting the importance of this approach by parents.

One area of identity affirmation parents recognized as lacking for their children was in models of nonbinary identities. The parents express an understanding that their child rarely, if at all, sees people who share that aspect of their identity. Bianca wanted her child to get more “exposure” to people who “exist outside of this rigid gender binary,” and as Christine points out, “they see plenty of cisgender people. I’m not worried about them not having cisgender role models.” As a result, parents take steps to correct this. Christine took a book that did not explicitly address nonbinary experiences and “spent six hours whiting out and writing new words over every part of it.” Adrienne started going out of her way to attend a queer rock-climbing club in an effort to “cultivate a [queer] community” for her child, and introduced them to a nonbinary person she met there. Iris and Dahlia also made explicit efforts to provide their children with gender-diverse representation.

Same-gender models are understood to be important in one’s development and understanding of gender [55, 56]. While children imitate individuals with traits they identify with, and not just people of their gender [55], much research has reaffirmed the importance of same-gender models. Although these studies have generally focused on girls and young women, research from nonbinary author Koonce [57] states from their professional experience “.it is in the mirroring of others that [non-binary identities] truly take form” (location 3,021). Kuper and colleagues [56] add that exposure to models with diverse gender presentations is crucial in supporting ongoing gender exploration.

Parents come to understand that the act of negotiating one’s way through transphobic and cisnormative systems is a “heavy lift” (Bianca). They do not want their child to have to be “constantly” (Bianca) educating and correcting people, especially “[not] by themselves” (Christine). Parents would rather take on the “forefronting” (Dahlia) and “emotional labor” (Christine) themselves. The overwhelming sentiment from parents is that “[their child] should not be expected to do” (Grace) the work of making space for themselves.

Despite the diverse realities of the children and experiences of the parents, a pattern emerged: parents respected their child’s ability to know and communicate their desires and leveraged their abilities and resources as adults to support them. A common example involved pronouns in public. According to Garcia [58], ‘gender math’ refers to the complex calculations parents must make when attempting to prioritize their child’s well-being while simultaneously accounting for restrictive systems of cisnormativity. For decisions regarding sharing their child’s gender identity, Bianca and her child devised a collaborative scale-ranking system to gauge the significance of individuals. “Is this an important person that we need to [understand your gender]? Is this person not worth it? Can this person potentially be toxic?” In similar fashion, Emiko and her child “came up with a system” to organize how much they care about the person in question, and gave us the example, “Do we care about the gas station attendant? Not really.” Powell and colleagues [42] similarly found that sharing the child’s gender with extended family was “often led by the child” (p.4). Additionally, a parent in their study shared that when they had made the decision without their child’s consent it “caused a big problem” (p.4). This kind of collaborative approach empowers the child to make decisions while also providing the assurance of a supportive caregiver during challenging circumstances.

In situations where the child was subject to explicit invalidation and/or transphobia, parents very readily took strong action. Adrienne shared that her child’s grandma refused to correctly gender the child. In response, she “nearly asked her to leave,” and was currently undergoing a “grandma rehabilitation plan.” Adrienne made sure it was clear to both the grandparent and the child that transphobic behavior was “[not] welcome under this roof.” Christine and Grace shared instances of invalidating behavior by their child’s classmates relating to gender which prompted them both to contact their child’s teachers, demanding that they “do better” (Christine) to make their child feel safe and included in the classroom. Ehrensaft [59] advocates for parental involvement in actively dismantling social pathologies that adversely affect trans youth such as gender policing and harassment. She recommends methods such as direct intervention within broader institutional spheres, encompassing schools, social institutions, and policy-making bodies.

While strategies were initially discussed between the parent and child, many parents became exceptionally proactive in their support. The parents’ efforts extended beyond casual conversations. While correction after an instance of misgendering or other aggression is crucial, the parents recognized the importance of minimizing these experiences before they occur. Bianca decided to coach her child’s soccer team “because [they] wanted to make sure [the team] was a safe space [for their child]” and that all the children “could hear role modeling of using ‘they’ [pronouns].” Emiko reached out to her child’s teachers and sports coaches, urging them to adopt inclusive language and suggesting alternatives to gendered terms: “You could use, ‘alright players,’ or ‘alright team.’” Fiona found a “How to They/Them book” immensely helpful, sent it to everyone in her family and is “making [them] read it.” Christine and her husband expended “a lot of emotional labor” to get their family on the “right page.” She shares that advocating for her child has been “a huge time investment on [her] part,” but that she is “fighting the good fight,” echoing a sentiment shared by all parents.

Theme 4: Gender is just one aspect of who my child is.

In society, there’s a prevalent notion that undergoing a gender ‘transition’ entails a departure from one’s ‘pre-transition’ self. This misconception is especially pronounced in expectations regarding clothing and presentation, where there is a common belief that AMAB individuals who are TNB must present as feminine, and vice versa. However, this presumption extends beyond outward appearance to encompass behavior and preferences as well. It originates from the entrenched concept of gender binary, whereby deviation from assigned gender norms is often perceived as a desire to conform to the norms of the ‘opposite gender.’ Through the parents’ demonstrated willingness to let their child’s expressed emotions and demonstrated actions alter their preconceived notions and internalized frameworks, the parents came to understand that very little about their child’s ‘being’ changed after sharing their nonbinary identity [2, 42]. These experiences helped the parents expand their understanding of what gender is, but also what gender is not: a determining characteristic of a person’s identity.

In Grace’s experience, her child’s nonbinary identification “did not change anything about [their] clothes or identity or books or anything.” In comparing her child pre- and post-nonbinary identification, Adrienne explains “they wear the same things they’ve always worn. The main [difference] is they tell people that they’re nonbinary.” Instead of basing presentation off of gender, Bianca observed her child dressing “for practical reasons,” namely the weather and season, and Helena shared her child’s understanding of clothes being as plain as “[these shoes] are on my feet and I’m wearing them.” Fiona shared that at first when learning that her AMAB child was nonbinary, her brain struggled holding it as they felt that her child “seem[ed] like such a boy.” Fiona had to open her mind to what nonbinary could mean through meeting people of diverse gender identities and presentations, and reading books, and arrived at the conclusion that “if there are 7 billion people, [there are] 7 billion [gender] identities.”

A big takeaway explicitly communicated by six parents, is that gender is just a part of how they understand their child as a person. Powell and colleagues [42] had the same finding with parents of TNB children between the ages of 10 and 18. Parents acknowledge that while the identification with naming a nonbinary identity (nonbinary, boy-girl, etc.) holds personal, practical, and political significance, and while it can be and is a very important identity to many people, it does not provide a singular definition of their child. Iris identifies her child’s nonbinary identity as just “another characteristic, [but] not what defines [their child].” Dahlia adds that “[gender] is a piece of information, but it’s not the most interesting or important thing about who they are.” Christine understands the nonbinary identity to be a component of their child’s identity in addition to “loving Lego and loving dresses, and jumping off stuff, and hitting stuff with other stuff. It’s just part of their kernel, you know?” Expressions like the highlighted quote are not meant to invalidate or diminish the nonbinary experience. Instead, they seek to understand gender as an integral aspect of the child’s being, one that significantly influences their interactions within society. Nevertheless, this acknowledgment does not necessitate a complete overhaul of their core identity. In reflecting, Iris shares, “I try to think of them as a person that I’m finding out about and not a set of expectations,” and that she tries to “not to make [gender] a big deal […] a guiding thing that I have taken from [my child].” Adrienne adds, “It’s the same child that I’ve always known, just using different pronouns,” and Bianca is glad that her child feels that “[they] can be anything [they] want. But [does not] have to be not something.” These parents came to recognize that their child’s nonbinary gender was simply an extension of who the child already was. Bianca found this experience “really joyous,” and for Helena, seeing their child being themself was “indescribably heartwarming.” Dahlia feels that it is “such a gift,” and “rewarding to know that they know” they can be their true self, and Fiona echoes that she feels “happy… that they’ve discovered that this can be their identity.”

## General discussion

We examined how parents of nonbinary children learn to support their child through the lenses of ontological relativism and epistemological constructivism. We interpreted these parents’ experiences, taking on the role of ‘subjective storytellers’ [48]. Despite space limitations, our aim was to offer a comprehensive exploration and contribute nuanced insights to the limited, yet growing, understanding of parental support for gender affirmation among TNB youth [6, 13, 14, 25, 31, 47, 52]. Our aim was not merely to ‘fill a gap’ but to enrich the broader understanding that we and others are collectively developing [48]. Our strength lay in adhering to a ‘Big Q’ approach that challenges the structured and positivist paths towards absolutisms.

As a result, RTA was the perfect methodology for our study. We were interested in “…process and meaning, over cause and effect; a critical and questioning approach to life and knowledge; the ability to reflect on the dominant assumptions embedded in [our] cultural context—being a cultural commentator as well as a cultural member; the ability to read and listen to data actively and analytically […] a desire for understanding that is about nuance, complexity, and even contradiction, rather than finding a nice tidy explanation…” ( [48], loc. 1334). We made active efforts to reflect on dominant assumptions and divest from cisnormativity in our daily lives [35]. Moreover, we, the authors–cultural commentators– are still learning what it means to be trans, genderqueer, and nonbinary–cultural members– in a society that has traditionally only had space for men and women. We are collectively working on this tapestry of understanding.

While all parents supported their child’s gender identity, some struggled with doubts and concerns, highlighting the nuances and even contradictions in their experiences. Parents faced a complex situation with no clear guidance on raising nonbinary children, amidst widespread fear-mongering targeting parents of TNB children [47, 53]. Although we focus on parents and avoid pathologizing and stigmatizing rhetoric, it is a matter of fact that TNB children are facing intense violence and legislative attacks, with more anti-trans laws introduced in the past nine years than in the previous 240, aiming to restrict their healthcare, legal recognition, participation in school activities, and more [9, 12, 60]. In the face of this anti-trans violence and exclusionary legislation, recent scholarship emphasizes that trans communities extend beyond struggle and hardship, embracing resilience and thriving through radical hope [47, 61–63].

de Bres [41]’s recommendations fit well within this call to shift the narrative towards joy. This is why we aimed to ask similar questions and create a realistic but uplifting account that acknowledges struggles but celebrates the joys of parenting a nonbinary child. As de Bres [41] reminds us, the questions researchers ask shape the responses they receive. Common questions like ‘When did you first notice your child was gender-diverse?’ often prompts a ‘coming-out’ narrative. Shifting to asking questions such as ‘What has been the most rewarding part of raising a [preferred term] child?’ can prompt reflection through a more joy- and strengths-based lens.

One of our primary insights was the discovery that parents approached parenting a nonbinary child within minimal preconceptions. This finding was surprising, considering prevalent societal narratives, often steeped in fearmongering as previously discussed. While we recognize that not all initial parental reactions may have been disclosed during interviews, those that were shared demonstrated a nuanced understanding of nonbinary identities. Parents deconstructed much of society’s cisnormativity and debunked transphobic misconceptions. Particularly notable was their collective sentiment that their child’s nonbinary identity is ‘just’ simply another integral aspect, as natural as their love for activities like soccer or building with Legos, and as natural as their other children identifying as girls or boys.

Furthermore, we were impressed by their comprehension of the fluidity inherent in their children’s nonbinary identities, embracing expressions that may encompass elements considered traditionally masculine or feminine. This included recognizing that their child’s desire to wear a dress and paint their nails on one day and wear pants and a t-shirt another is not merely an ‘exploration’ but a genuine expression of their gender.

We were heartened by the rapid evolution in societal acceptance of genderqueer identities in recent years. As early as 2020, during our collective virtual interactions due to the COVID-19 pandemic, we began to regularly encounter individuals displaying pronouns next to their names -a small but significant shift. Four years later we observe a growing recognition and understanding of ‘they/them’ pronouns, alongside remarkable parental support for young children in the Northeast who identify as nonbinary. Beyond just gender, this progress inspires hope for a world that embraces ambiguity and rejects rigid absolutes, celebrating the diverse spectrum of human experience rather than confining individuals to either one thing or another.

Throughout our exploration of parental experiences, we were struck by their responsiveness to their children’s desires and needs. While following a child’s lead is not a new concept, these parents had minimal pre-existing knowledge of TNB experiences. Their support required a leap into uncharted territory, yet with open minds and attentive listening, they made decisions that appeared highly supportive and affirming of their child’s gender identity to us. Witnessing this support brought us profound joy and optimism amidst pervasive fear mongering rhetoric and transphobic narratives.

Research consistently highlights improved outcomes for TNB individuals when their families support their gender identities. While the relationship between familial support and wellbeing is nuanced, it remains clear that supportive parenting plays a pivotal role. Even within a transphobic and cisnormative society that often lacks understanding of pronoun usage and genderqueer identities, and remains fixated on binary norms, these parents demonstrate that by valuing their nonbinary children’s communications of needs, they can profoundly make them feel seen, loved, and supported.

In conclusion, our pioneering study focuses uniquely on the experiences of parents of nonbinary children, applying rigorous ‘Big Q’ principles and emphasizing narratives of joy. Utilizing RTA, we hope to contribute valuable insights to understanding and supporting young nonbinary children and their families. Christine’s words resonate deeply: “Sometimes I just say to my husband, ‘We’re doing it, this child’s heart is intact. This child’s heart is strong, and intact. And, no matter what, we’re doing it right if their heart is intact’.” This sentiment underscores our commitment to providing shared experiences and celebrating the resilience of TNB communities throughout our research. Our work adds to the growing body of research aimed at promoting understanding and support for nonbinary individuals.

We hope our findings contribute to the shift away from adult-centric perspectives and towards respecting children’s ability to be cognizant of their own needs, as well as understand themselves in the context of broader society. As our study shows, children will be who they are, and will express themselves, despite any barriers. Banning learning or restricting ideas does not control children; it only harms them. Violence against children should never be normalized. Families and youth deserve legal autonomy, and everyone should be educated about the diversity that exists in this world. We are optimistic that these experiences will inspire advocates and lawmakers to recognize children as experts in their own lives, as the parents in our study did.

Our research highlights how parents can deconstruct binary conceptions of gender in favor of more open-minded perspectives, positively impacting both family dynamics and children’s well-being. We are hopeful that these narratives will encourage adults who work with children -therapists, social workers, teachers, coaches, pediatricians, and others- to re-examine their own understanding of gender. To parents who may be struggling or worried about their nonbinary child: we hope our work offers guidance and hope. The gender binary can be unlearned, and new pronouns can be practiced. You can learn from your child and from the growing resources available. Your child knows themself. You will continue to learn about them and their identity, just as they do, and just as every person does.

## Data Availability

The authors confirm that the data supporting the findings of this study are available within the article. Due to the sensitive nature of the research and limitations of participant consent, the supporting data is not available for sharing beyond what is presented in the article.

## Abbreviations

LGBTQIA+: lesbian, gay, bisexual, transgender, queer or questioning, intersex, and sexual. The + represent other identities that are noy included in the acronym
TBN: Transgender and nonbinary
RTA: Reflexive Thematic Analysis
TGD: Transgender and Gender Diverse
AMAB: Assigned Male At Birth
AFAB: Assigned Female At Birth

## Footnotes

1 In qualitative research “… you are *not* seeking to show that you have found an empty cell in the spread-sheet of ultimate truth about the topic, which your study will fill in. We think it’s useful to get beyond the filling the gap idea, and conceptualize our qualitative analyses as contributing something to a rich tapestry of understanding that we and others are collectively working on, in different places, spaces and times” (Braun & Clarke, 2022, loc.5192).

2 The term ‘method’ risks becoming “...a practically orientated descriptive summary, rather than a more theoretically-oriented and reflexive discussion of what, why and how one did the research” (Braun & Clarke, 2022, loc.5232).

3 “How Do Children Identifying Beyond the Gender Binary and Their Parents Understand Gender?” (IRB ID: CR-01-STUDY00002649).

4 We emphasize the significance of dynamic and reflective positionality in research, advocating to move beyond static researcher-centered perspectives towards amplifying marginalized communities (Salinas-Quiroz et al., 2024). We also recognize challenges posed by word limits and traditional research guidelines yet stress the continual integration of reflexivity to enrich qualitative inquiry.

5 Although we collected demographic data, it will not be fa central part of our analysis. The research on demographic factors related to TNB identities is limited, and our sample size is too small to draw significant conclusions about ‘potential correlations.’ Moreover, it’s important to consider broader historical contexts. While there is a common belief that white, wealthy individuals with higher education levels are more socially progressive, focusing solely on these factors overlooks how legal, medical, and academic institutions have systematically reinforced racial, gender and class hierarchies. Emphasizing these demographics risks overshadowing the richness of individual experiences, which is the true strengths of qualitative research.
